# Lifestyle Factors and Thromboembolic Risk in Atrial Fibrillation: Age‐Dependent Effects of Smoking and Alcohol Consumption

**DOI:** 10.1002/joa3.70413

**Published:** 2026-07-07

**Authors:** Masamichi Yano, Yasuyuki Egami, Mikako Kise, Taichi Mukai, Noriyuki Kobayashi, Ayako Sugino, Masaru Abe, Hiroaki Nohara, Shodai Kawanami, Koji Yasumoto, Naotaka Okamoto, Yasuharu Matsunaga‐Lee, Masami Nishino

**Affiliations:** ^1^ Division of Cardiology Osaka Rosai Hospital Sakai Osaka Japan

**Keywords:** alcohol consumption, atrial fibrillation, cardioembolic stroke, smoking

## Abstract

**Background:**

Atrial fibrillation (AF) relates to cardioembolic stroke (CS), but the role of lifestyle factors, including smoking and alcohol, remains unclear.

**Methods:**

This retrospective, nationwide, multicenter case–control study used the Inpatient Clinico‐Occupational Database of the Rosai Hospital Group from 2005 to 2022. Patients with AF and CS were identified using ICD‐10 codes. Smoking status and alcohol consumption were assessed at admission through structured interviews. We examined age‐stratified differences in smoking and alcohol consumption and evaluated the impact of smoking and alcohol intake on the risk of CS.

**Results:**

Among 26 915 patients, 5688 had AF‐related CS and 21 227 had AF without CS. Current smoking was more prevalent in AF patients with CS aged from 40–49 to 80–89 years than those without (all *p* < 0.001). Multivariable logistic regression showed smoking was independently associated with CS overall (all *p* < 0.001) and by age group, and daily alcohol intake was associated with CS overall and in patients ≤ 75 years (*p* = 0.021 and 0.044). The combined score of chronic heart failure, hypertension, age > 75‐year (weighted × 2), and diabetes mellitus (CHA_2_D, range 0–5) increased with age, from 0.66, 0.67, 1.36, 2.43, 2.83, and 2.94 (average) in the 40–49, 50–59, 60–69, 70–79, 80–89, and ≥ 90‐year groups (*p* < 0.001), whereas the prevalence of current smoking and daily alcohol consumption declined progressively with age (*p* < 0.001).

**Conclusions:**

Smoking and daily alcohol consumption associate with CS risk in AF patients, especially younger individuals with low CHA_2_D scores, highlighting the role of lifestyle in risk assessment.

## Graphical abstract

Association of current smoking and daily alcohol consumption with cardioembolic stroke in patients with atrial fibrillation.

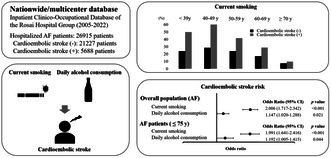

## 1 Introduction

Atrial fibrillation (AF) is one of the most prevalent cardiac arrhythmias and represents a substantial burden on healthcare systems worldwide [1]. The prevalence of AF is projected to rise further in parallel with global population aging. Stroke is a major and devastating complication of AF; approximately 25% of ischemic strokes are of cardioembolic origin, with AF being the leading underlying cause. Moreover, nonvalvular AF is associated with a fivefold increased risk of ischemic stroke [2]. Numerous studies have established several independent clinical risk factors for incident AF, including advanced age, hypertension, diabetes mellitus, congestive heart failure, coronary heart disease, and valvular heart disease [3]. Beyond these traditional risk factors, accumulating evidence suggests that lifestyle‐related factors play an important role in the development of AF. In particular, current smoking has been consistently associated with an increased incidence of AF, conferring more than a twofold higher risk compared with non‐smoking [4]. Moreover, alcohol consumption has been identified as a risk factor for AF, with prior studies demonstrating that even moderate alcohol intake is associated with an elevated risk of AF [5]. On the other hand, the CHA_2_DS_2_‐VASc or CHA_2_DS_2_‐VA scores are the most widely used models for predicting thromboembolic risk in patients with AF [1, 6]. However, lifestyle‐related factors, including smoking and alcohol consumption, are not included in these scoring systems. This may be because the association between smoking or alcohol consumption and thromboembolic events in AF has been investigated in previous studies, but consistent evidence supporting a clear relationship has been lacking, which likely explains why these factors were not incorporated into established risk scores. Although smoking and alcohol consumption have been proposed as potential risk factors for thromboembolism, their association with thromboembolic events in patients with AF remains controversial. Therefore, in the present study, using a large‐scale database of the Inpatient Clinico‐Occupational Database of Rosai Hospital Group (ICOD‐R) provided by 32 hospitals crossing the entire area of Japan, we examined the effects of current smoking and daily alcohol consumption among patients with AF‐related cardioembolic stroke (CS).

## 2 Methods

### 2.1 Study Population

This was a retrospective nationwide, multicenter, hospital‐based unmatched case–control study using data of the Inpatient Clinico‐Occupational Survey of the Rosai Hospital Group from 2005 to 2022. Details of this survey have been described elsewhere [7, 8]. Briefly, the Inpatient Clinico‐Occupational Survey simultaneously collected clinical and occupational history data for all inpatients aged 15 years or older admitted to facilities within the nationwide Rosai Hospital Group. The International Classification of Diseases, Tenth Revision (ICD‐10) codes were used to identify each disease [9]. Patient clinical histories were integrated with summaries of treatments documented by attending physicians. Each physician could record up to seven confirmed diagnoses, which were coded according to the ICD–10. Between 2005 and 2022, patients with AF‐related CS were defined by the presence of ICD‐10 codes for AF (I48.0 [paroxysmal AF], I48.1 [persistent AF], I48.2 [long‐standing AF] and I48.9 [AF and atrial flutter, unspecified]) in combination with cerebral infarction due to embolism of cerebral arteries (I63.4), and with available information on smoking and alcohol consumption. As a control group, patients hospitalized for AF during the same period were included if their primary diagnosis at admission was AF (I48.0, I48.1, I48.2 and I48.9), smoking and alcohol consumption data were available, and no diagnosis of cerebral infarction due to embolism (I63.4) was present. History of heart failure was defined based on ICD‐10 codes I50.x.

In the present study, we compared smoking status and alcohol consumption between AF patients with and without CS stratified by age. We also evaluated the impact of smoking and alcohol intake on the risk of CS and examined the age‐specific distributions of established thromboembolic risk factors, stratifying patients according to age ≤ 75 years and > 75 years, which corresponds to the commonly used cutoff for risk stratification in the CHADS_2_/CHA_2_DS_2_‐VASc scoring systems [6]. Thromboembolic risk factors were assessed using the CHA_2_D score, a non‐standard score including chronic heart failure (C), hypertension (H), age > 75 years (A, weighted ×2), and diabetes mellitus (D), excluding prior stroke due to the inability to distinguish it from the index cerebrovascular event in the ICD‐10 dataset. This study was approved by the ethics committees of Osaka Rosai Hospital (approved number: 2024–147).

### 2.2 Smoking and Alcohol Consumption

Smoking and alcohol consumption data were assessed at the time of hospital admission using structured interviews. Smoking frequency was categorized as never, former, and current. Drinking frequency of alcohol intake was categorized as never, former/current, and daily.

### 2.3 Statistical Analysis

JMP 17 statistical software (SAS Institute Inc., Cary, North Carolina, USA) was used for the statistical analysis. Continuous variables were expressed as the median [interquartile range]. A normality test was performed for continuous variables by a Shapiro–Wilk W test. A normal distribution was not confirmed for all variables. Categorical data were expressed as the number (percentage) and were compared using the chi‐square test. Multivariable logistic regression analysis for CS was performed using the factors including age, gender, height, body weight, current smoking, daily alcohol consumption, hypertension, and diabetes mellitus. Variance inflation factors (VIFs) were calculated to assess multicollinearity, with values < 5 indicating no significant multicollinearity [10]. In the overall and age‐stratified populations (≤ 75 and > 75 years), VIFs ranged from 1.020 to 3.413, all below 5, indicating no significant multicollinearity. Both height and body weight were included in the models. Odds ratios (ORs) and 95% confidence intervals were calculated and *p*‐value < 0.05 was considered significant.

## 3 Results

### 3.1 Patient Characteristics

Between 2005 and 2022, a total of 26 915 patients were enrolled. Of these, 5688 patients had AF with AF‐related CS, defined by the presence of ICD‐10 codes for AF (I48.0, I48.1, I48.2, and I48.9) and cerebral infarction due to embolism (I63.4). The remaining 21 227 patients were hospitalized with AF as the primary diagnosis, defined by the presence of ICD‐10 codes for AF (I48.0, I48.1, I48.2, and I48.9), and had no diagnosis of cerebral infarction due to embolism (I63.4). Patient characteristics are shown in Table 1. Patients with CS were significantly older than those without CS (median age, 81 [74–86] vs. 71 [64–78] years; *p* < 0.001). The proportion of males was lower in AF patients with CS (52.7%) compared with those without CS (65.3%), with a corresponding higher proportion of females (*p* < 0.001). Patients with CS had significantly shorter height (157 [150–165] vs. 164 [155–170] cm; *p* < 0.001) and lower body weight (54 [46–64] vs. 63 [54–72] kg; *p* < 0.001). The prevalence of hypertension was similar between the two groups (50.4% vs. 50.3%; *p* = 0.947). In contrast, diabetes mellitus was slightly more common in AF patients with CS than in those without CS (17.3% vs. 15.9%; *p* = 0.012).

**TABLE 1:** Baseline patient characteristics.

	AF without CS (*n* = 21 227)	AF with CS (*n* = 5688)	*p*
Age	71 [64, 78]	81 [74, 86]	< 0.001
Male/Female	13 851 (65.3)/7376 (34.8)	2996 (52.7)/2692 (47.3)	< 0.001
Height	164 [155, 170]	157 [150, 165]	< 0.001
Body weight	63 [54, 72]	54 [46, 64]	< 0.001
Smoking			
Former/Current	11 280 (53.1)	2247 (39.5)	< 0.001
Current (daily)	2756 (13.9)	740 (13.0)	0.958
Drinking			
Former/Current	12 927 (60.9)	2767 (48.6)	< 0.001
Current (daily)	6681 (31.5)	1362 (24.0)	< 0.001
History of heart failure	827 (14.5)	3499 (16.5)	< 0.001
Hypertension	10 685 (50.3)	2866 (50.4)	0.947
Diabetes mellitus	3368 (15.9)	981 (17.3)	0.012
Dyslipidemia	3036 (14.3)	632 (11.1)	< 0.001
Hyperuricemia	1281 (6.0)	227 (4.0)	< 0.001

*Note:* Continuous data are presented as the median (interquartile range). Categorical variables are presented as numbers (percentage).

Abbreviations: AF, atrial fibrillation; CS, cardioembolic stroke.

### 3.2 Association Between Smoking Status, Age, and Cardioembolic Stroke

**FIGURE 1:**
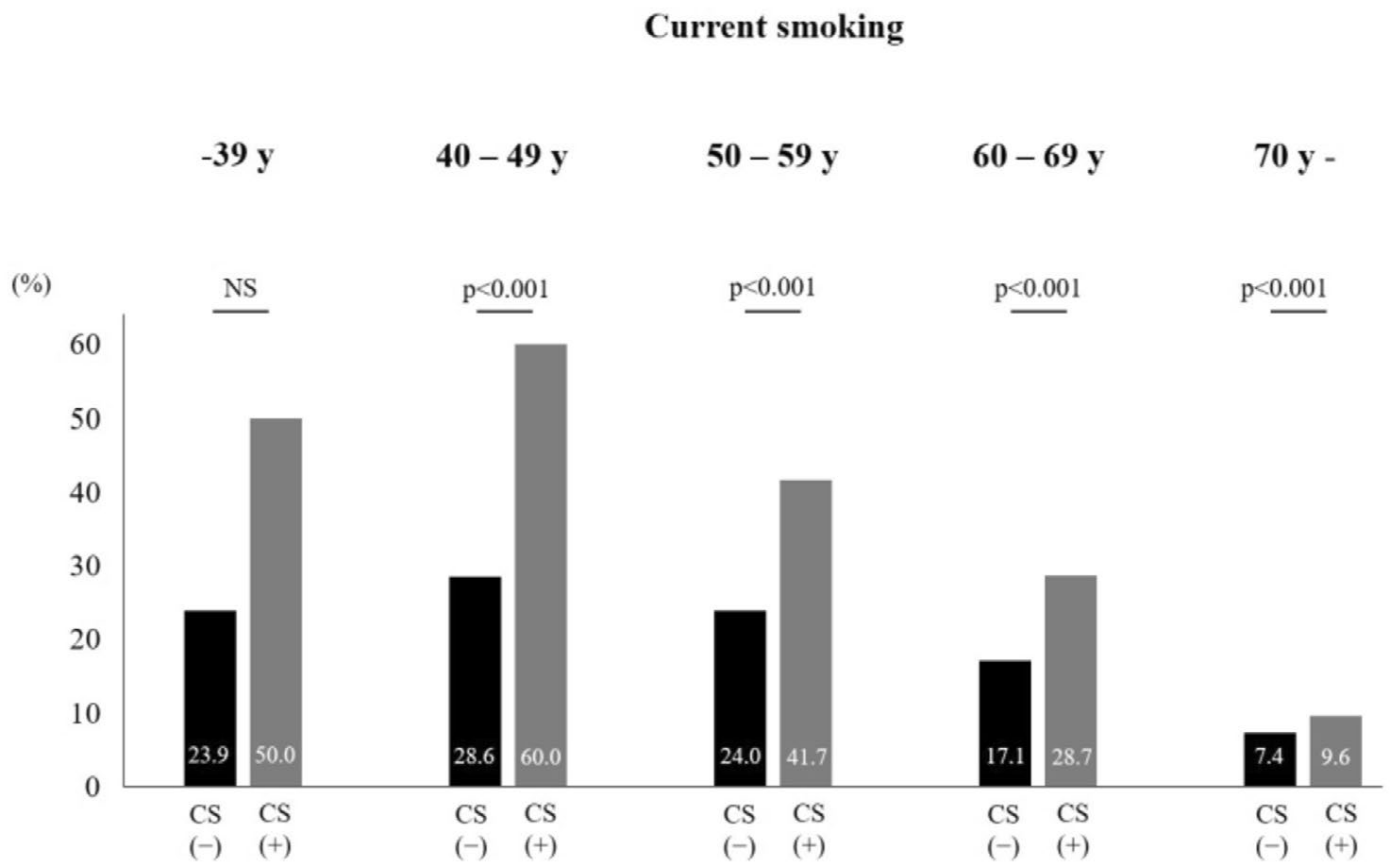
Age‐stratified prevalence of current smoking in AF patients with and without CS. AF, atrial fibrillation; CS, cardioembolic stroke; NS, not significant.

Age and sex‐stratified comparisons of smoking status according to the presence or absence of CS are presented in Table 2. In both AF patients with and without CS, the proportions of former or current smokers and current daily smokers decreased significantly with advancing age (*p*‐for trend < 0.001 for both). No significant differences between AF patients with and without CS were observed for former or current smoking. In contrast, the prevalence of current smoking was significantly higher in AF patients with CS compared with those without CS in younger, middle‐aged, and older patients ([40–49], [50–59], [60–69], [70–79], [80–89] years; *p* < 0.001 for all) (Table 2 and Figure 1). Sex‐stratified analyses demonstrated that, across all age categories, males consistently had higher prevalences of former/current smoking and current smoking than females, regardless of CS status (*p* < 0.001).

**TABLE 2:** Age‐stratified smoking and drinking status in patients with and without cardioembolic stroke.

AF without CS (*n* = 21 227) (Male:13851, Female:7376), AF with CS (*n* = 5688) (Male:2996, Female:2692)	−39 years, CS (−): *n* = 243, CS (+): *n* = 2	40–49 years, CS (−): *n* = 914, CS (+): *n* = 30	50–59 years, CS (−): *n* = 2459, CS (+): *n* = 151	60–69 years, CS (−): *n* = 5573, CS (+): *n* = 676	70–79 years, CS (−): *n* = 7840, CS (+): *n* = 1739	80–89 years, CS (−): *n* = 3697, CS (+): *n* = 2272	90 years, CS (−): *n* = 501, CS (+): *n* = 818
Smoking							
Former							
AF without CS							
Total	125 (51.4)	591 (64.7)	1621 (65.9)	3518 (63.1)	3946 (50.3)	1373 (37.1)	106 (21.6)
Male	118 (55.1)	546 (71.1)	1479 (73.8)	3138 (77.5)	3455 (71.6)	1202 (65.3)	82 (55.4)
Female	7 (24.1)	45 (30.8)	142 (31.2)	380 (25.0)	491 (16.3)	171 (9.2)	24 (6.8)
AF with CS							
Total	2 (100.0)	22 (73.3)	106 (70.2)	423 (62.6)	853 (49.1)	723 (31.8)	118 (14.4)
Male	2 (100.0)	20 (74.1)	92 (78.0)	388 (75.1)	783 (69.6)	645 (63.6)	93 (48.2)
Female	0 (0)	2 (66.7)	14 (42.4)	35 (22.0)	70 (11.4)	78 (6.2)	25 (4.0)
Current							
AF without CS							
Total	58 (23.9)	261 (28.6)	590 (24.0)	953 (17.1)	703 (9.0)	175 (4.7)	16 (3.2)
Male	54 (25.2)	237 (30.9)	528 (26.4)	821 (20.3)	591 (12.3)	134 (7.3)	10 (6.8)
Female	4 (13.8)	24 (16.4)	62 (13.6)	132 (8.7)	112 (3.7)	41 (2.2)	6 (1.7)
AF with CS							
Total	1 (50.0)	18 (60.0)	63 (41.7)	194 (28.7)	289 (16.6)	160 (7.0)	15 (1.8)
Male	1 (50.0)	16 (59.3)	55 (46.6)	175 (33.9)	254 (22.6)	137 (13.5)	11 (5.7)
Female	0 (0)	2 (66.7)	8 (24.2)	19 (12.0)	35 (5.7)	23 (1.8)	4 (0.6)
Drinking							
Former/Current							
AF without CS							
Total	176 (72.4)	692 (75.7)	1909 (77.6)	3942 (70.7)	4550 (58.0)	1548 (41.9)	110 (22.0)
Male	161 (75.2)	614 (80.0)	1682 (83.9)	3328 (82.2)	3727 (77.3)	1259 (68.4)	77 (52.0)
Female	15 (51.7)	78 (53.4)	227 (49.9)	614 (40.3)	823 (27.3)	289 (15.6)	33 (9.4)
AF with CS							
Total	1 (50.0)	25 (83.3)	115 (76.2)	474 (70.1)	1080 (62.1)	922 (40.6)	150 (18.3)
Male	1 (50.0)	23 (85.2)	98 (83.1)	426 (82.4)	923 (82.0)	732 (72.2)	109 (56.5)
Female	0 (0)	2 (66.7)	17 (51.5)	48 (30.2)	157 (25.6)	190 (15.1)	41 (6.6)
Daily							
AF without CS							
Total	56 (23.0)	356 (38.9)	1085 (44.1)	2254 (40.4)	2292 (29.2)	599 (16.2)	39 (7.8)
Male	50 (23.4)	331 (43.1)	1015 (50.7)	2024 (50.0)	2055 (42.6)	536 (29.1)	34 (23.0)
Female	6 (20.7)	25 (17.1)	70 (15.4)	230 (15.1)	237 (7.9)	63 (3.4)	5 (1.4)
AF with CS							
Total	1 (50.0)	13 (43.3)	74 (49.0)	293 (43.3)	572 (32.9)	364 (16.0)	45 (5.5)
Male	1 (50.0)	12 (44.4)	69 (58.5)	282 (54.6)	537 (47.7)	329 (32.5)	39 (20.2)
Female	0 (0)	1 (33.3)	5 (15.2)	11 (6.9)	35 (5.7)	35 (2.8)	6 (1.0)

*Note:* Categorical variables are presented as numbers (percentage).

Abbreviations: AF, atrial fibrillation; CS, cardioembolic stroke.

### 3.3 Association Between Alcohol Consumption Status, Age, and Cardioembolic Stroke

**FIGURE 2:**
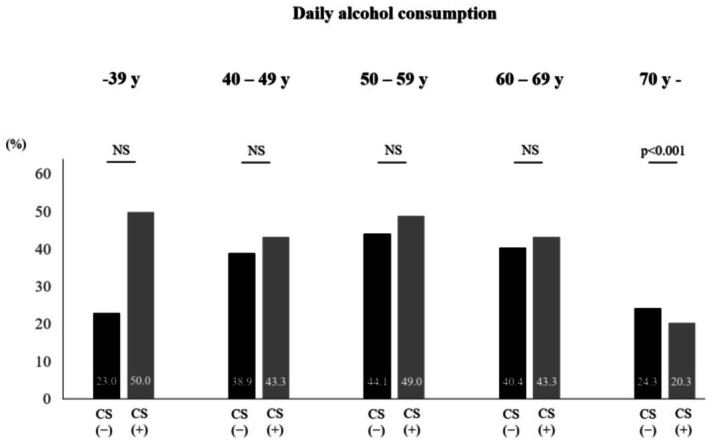
Age‐stratified prevalence of daily alcohol consumption in AF patients with and without CS. AF, atrial fibrillation; CS, cardioembolic stroke; NS, not significant.

Age‐stratified comparisons of alcohol consumption according to the presence or absence of CS are presented in Table 2. In both AF patients with and without CS, the proportions of former or current drinkers and daily alcohol consumers decreased significantly with advancing age (*p* for trend < 0.001 for both). No significant differences between AF patients with and without CS were observed for former or current drinkers in any age category. Similarly, the prevalence of current drinkers did not differ significantly between AF patients with and without CS across age categories (Figure 2). Sex‐stratified analyses demonstrated that, across all age categories, males consistently had higher prevalences of former/current alcohol consumption than females, regardless of CS status (*p* < 0.001).

### 3.4 Determinants of Cardioembolic Stroke in Patients With Atrial Fibrillation

**FIGURE 3:**
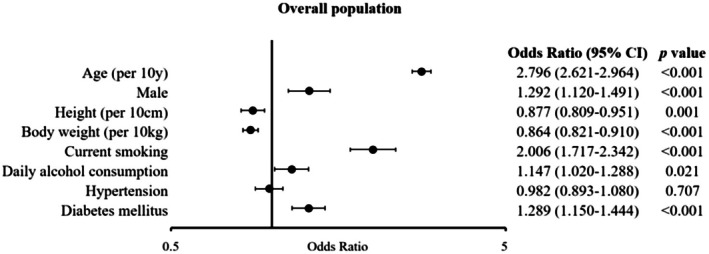
Multivariable logistic regression analysis for CS in overall population illustrated as Forest plot. CI, confidence interval; CS, cardioembolic stroke.

**FIGURE 4:**
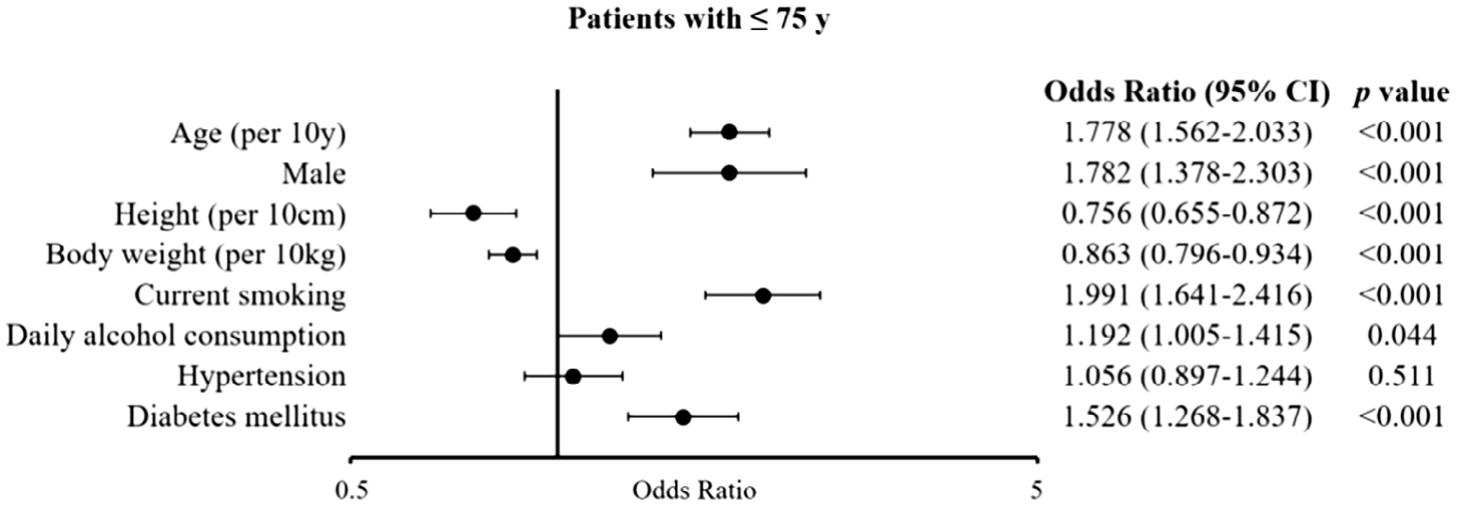
Multivariable logistic regression analysis for CS in patients with ≤ 75 years illustrated as Forest plot. CI, confidence interval; CS, cardioembolic stroke.

**FIGURE 5:**
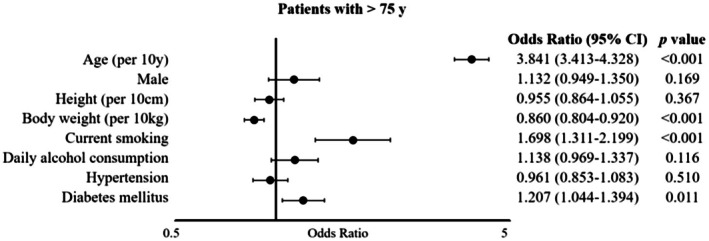
Multivariable logistic regression analysis for CS in patients with > 75 years illustrated as Forest plot. CI, confidence interval; CS, cardioembolic stroke.

To identify independent predictors of CS, we performed multivariable logistic regression analysis including cardiovascular risk factors and lifestyle variables (current smoking and daily alcohol consumption). The results of the multivariable logistic regression analysis for the overall population are presented in a forest plot in Figure 3. Advanced age, male sex, shorter height, lower body weight, current smoking, daily alcohol consumption, and diabetes were identified as independent and significant predictors of CS in overall population (*p* < 0.001, *p* < 0.001, *p* = 0.001, *p* < 0.001, *p* < 0.001, *p* = 0.021, and *p* < 0.001, respectively). In patients aged ≤ 75 years (*n* = 15 807), multivariable logistic regression analysis showed a similar pattern: advanced age, male sex, shorter height, lower body weight, current smoking, daily alcohol consumption, and diabetes were independent and significant predictors of CS (*p* < 0.001, *p* < 0.001, *p* < 0.001, *p* < 0.001, *p* < 0.001, *p* = 0.044, and *p* < 0.001, respectively) (Figure 4). In patients aged > 75 years (*n* = 11 108), multivariable logistic regression analysis demonstrated that advanced age, lower body weight, current smoking, and diabetes were independent and significant predictors of CS (*p* < 0.001, *p* < 0.001, *p* < 0.001, *p* < 0.001, and *p* = 0.011, respectively) (Figure 5).

### 3.5 Age‐Stratified Distribution of Heart Failure/Hypertension/Age/Diabetes Mellitus, Smoking, and Alcohol Consumption

The CHA_2_D score, along with the prevalence of current smoking and daily alcohol consumption among patients with AF and CS is shown in Table 3. The CHA_2_D score, a combined measure of chronic heart failure, hypertension, age > 75 years (weighted × 2), and diabetes mellitus (range 0–5), increased progressively with advancing age, with average scores of 0.66, 0.67, 1.36, 2.43, 2.83, and 2.94 in the 40–49, 50–59, 60–69, 70–79, 80–89, and ≥ 90‐year groups, respectively (*p* < 0.001). In younger patients, particularly those aged ≤ 59 years, the majority had low CHA_2_D scores (0–1), indicating a low burden of conventional thromboembolic risk factors. In contrast, higher CHA_2_D scores (≥ 2) were increasingly prevalent in older age groups, with a marked rise observed among patients aged ≥ 70 years. Despite the low CHA_2_D scores in younger age groups, who would conventionally be considered at lower risk for CS, the prevalence of current smoking and daily alcohol consumption was notably high. More than 40%–60% of patients aged ≤ 49 years were current smokers, and approximately 40%–50% reported daily alcohol consumption. Both smoking and daily alcohol use declined steadily with increasing age, paralleling the progressive increase in CHA_2_D scores (*p* < 0.001).

**TABLE 3:** Combined score of chronic heart failure, hypertension, age > 75 years (weighted × 2), and diabetes mellitus in patients with CS.

	−39 years (*n* = 2)	40–49 years (*n* = 30)	50–59 years (*n* = 151)	60–69 years (*n* = 676)	70–79 years (*n* = 1739)	80–89 years (*n* = 2272)	90 years (*n* = 818)
Heart failure							
Hypertension							
Diabetes mellitus							
CHA_2_D score							
Score 0	2 (100.0)	17 (56.7)	79 (52.3)	112 (16.6)	0 (0)	0 (0)	0 (0)
Score 1	0 (0)	7 (23.3)	48 (31.8)	286 (42.3)	280 (16.1)	0 (0)	0 (0)
Score 2	0 (0)	5 (16.7)	20 (13.3)	205 (30.3)	666 (38.3)	835 (36.8)	252 (30.8)
Score 3	0 (0)	1 (3.3)	4 (2.7)	70 (10.4)	592 (34.0)	1045 (46.0)	384 (46.9)
Score 4	0 (0)	0 (0)	0 (0)	3 (0.4)	173 (10.0)	345 (15.2)	164 (20.1)
Score 5	0 (0)	0 (0)	0 (0)	0 (0)	28 (1.6)	47 (2.1)	18 (2.2)
Average score	0	0.66	0.67	1.36	2.43	2.83	2.94
Current smoker	1 (50.0)	18 (60.0)	63 (41.7)	194 (28.7)	289 (16.6)	160 (7.0)	15 (1.8)
Daily alcohol consumption	1 (50.0)	13 (43.3)	74 (49.0)	293 (43.3)	572 (32.9)	364 (16.0)	45 (5.5)

*Note:* Categorical variables are presented as numbers (percentage).

Abbreviation: CS, cardioembolic stroke.

## 4 Discussion

### 4.1 Main Findings

In this large cohort of AF patients, several important findings emerged. First, smoking prevalence decreased progressively with advancing age in AF patients with and without CS; however, current smoking was consistently more prevalent in AF patients with CS than in those without CS across a wide range of age categories. This association was particularly evident in younger and middle‐aged patients and was observed in both sexes. Second, multivariable analyses identified advanced age, male sex, shorter height, lower body weight, current smoking, daily alcohol consumption, and diabetes as independent predictors of CS in the overall cohort. These associations were largely consistent in patients aged ≤ 75 years. In contrast, among patients aged > 75 years, advanced age, lower body weight, current smoking, and diabetes remained significant predictors, whereas sex, height, and daily alcohol consumption were no longer independently associated with CS. Accordingly, these findings suggest that lifestyle factors, particularly current smoking and alcohol consumption, are independently associated with CS in AF patients, with their relative contributions differing according to age.

### 4.2 Association Between Smoking and Cardioembolic Stroke in Patients With Atrial Fibrillation

Across all age groups, current smoking was substantially more prevalent among AF patients with CS than in the general population, as reported in the 2022 National Health and Nutrition Survey (≤ 39 years: 50.0% vs. 16.7%; 40–49 years: 60.0% vs. 20.8%; 50–59 years: 41.7% vs. 20.9%; 60–69 years: 28.7% vs. 17.3%; ≥ 70 years: 9.6% vs. 8.8%) [11]. Given the high prevalence of current smoking among AF patients, we next examined its potential impact on clinical outcomes, particularly CS, in our cohort. The relationship between smoking and clinical events in AF patients remains controversial. These findings should be interpreted in the context of previous studies, in which the association between smoking and stroke has been reported but remains inconsistent. Differences in study design and population, including the use of an inpatient database in the present study, may partly account for these discrepancies. Previous meta‐analyses have suggested that smoking does not significantly contribute to the risk of stroke or thromboembolic events in AF patients; however, it is associated with increased risks of all‐cause mortality, cardiovascular mortality, and major bleeding among patients receiving anticoagulant therapy [12]. On the other hand, in another large‐scale cohort study, AF patients who continued smoking after the diagnosis of AF had a higher risk of ischemic stroke and all‐cause mortality compared with those who quit smoking. In contrast, smoking cessation after the diagnosis of AF was associated with an approximately 30% reduction in the risk of ischemic stroke, as well as a substantial decrease in the risk of fatal stroke [13]. One possible explanation for these discrepant findings is the difference in the age distribution of smokers. In our cohort, the highest number of patients with CS was observed in the 70–79‐year age group, whereas the prevalence of current smoking increased sharply in patients aged ≤ 69 years. In multivariable analysis, current smoking was an independent risk factor for CS in the overall population; however, the effect was more pronounced in younger patients, with an OR of 1.991 in those aged ≤ 75 years compared with an OR of 1.698 in patients > 75 years (Figures 4 and 5). In older patients, the higher prevalence of lifestyle‐related diseases and other atherogenic risk factors may attenuate the relative impact of smoking on CS risk. Cigarette smoking promotes atherothrombosis through multiple mechanisms. It induces endothelial dysfunction and chronic vascular inflammation via oxidative stress, reduced nitric oxide bioavailability, and upregulation of inflammatory mediators, thereby increasing the activity of inflammatory cells and adhesion molecules [14]. Loss of normal endothelial function disrupts haemostatic balance and alters platelet physiology, contributing to thrombus formation [15]. In addition, smoking enhances platelet aggregability, elevates pro‐aggregatory mediators such as thromboxane A_2_, and inhibits fibrinolytic activity, further promoting thrombogenesis [16]. In the present study, in patients aged ≤ 59 years, the prevalence of current smoking exceeded 70%, whereas the prevalence of comorbidities such as hypertension and diabetes was relatively low. Despite the low burden of traditional cardiovascular risk factors, the occurrence of thromboembolic events in this population may reflect a prothrombotic effect of smoking. This study used an inpatient case–control design, which may introduce selection bias related to hospitalization and differences in prior clinical management. To indirectly evaluate the potential influence of catheter ablation‐related patient selection, we compared smoking and alcohol consumption patterns between institutions performing AF catheter ablation and those not performing ablation (Table S1). Smoking and alcohol consumption were not lower in ablation‐performing institutions, and among patients with CS, the prevalences of daily smoking and daily alcohol consumption were similar regardless of institutional ablation status. Furthermore, stratified multivariable analyses demonstrated that both current smoking and daily alcohol consumption remained independently associated with CS regardless of institutional AF catheter ablation status (Figures S1 and S2). It should also be noted that the strength of the association between current smoking and CS differed somewhat across institutional strata, with a higher odds ratio in ablation‐performing institutions (OR 2.20) than in non‐ablation institutions (OR 1.43). Although both associations were statistically significant, this heterogeneity suggests that the magnitude of the observed association may be influenced in part by differences in patient composition related to the reason for admission—including the possible inclusion in the control group of patients admitted for elective rhythm‐control procedures, who may have undergone lifestyle modification such as smoking cessation and reduction of alcohol intake as part of AF management. This interpretation is consistent with the predominantly null findings of prior studies [12], in which a positive association was largely confined to specific settings such as a pooled analysis of randomized trials or an analysis using a composite endpoint of stroke or death [6, 17], and with the observation that the association of smoking with stroke has been most evident in newly diagnosed, largely untreated patients [13]. Accordingly, the present associations should be regarded as hypothesis‐generating, and the possibility that they partly reflect selection related to the admission pathway, rather than a direct causal effect of smoking or alcohol on thromboembolism, should be kept in mind. These factors could have influenced both smoking status and the occurrence of CS. Therefore, the observed association should be interpreted with caution and does not imply a causal relationship.

### 4.3 Alcohol Intake and Risk of Cardioembolic Stroke in Patients With Atrial Fibrillation

Across all age groups, daily alcohol consumption was substantially more prevalent among AF patients with CS than in the general population, as reported in the 2022 National Health and Nutrition Survey (≤ 39 years: 50.0% vs. 19.4%; 40–49 years: 43.3% vs. 29.3%; 50–59 years: 49.0% vs. 27.1%; 60–69 years: 43.3% vs. 21.2%; ≥ 70 years: 20.3% vs. 10.5%) [11]. These population‐based observations prompted us to further investigate the association between daily alcohol consumption and CS in our cohort of patients with AF. In the present analysis, across a broad range of age categories from ≤ 39 to 70–79 years, the prevalence of daily alcohol consumption tended to be higher in patients with CS than in those without CS (Table 2). Furthermore, in multivariable logistic regression analyses, daily alcohol consumption was identified as an independent predictor of CS in the overall study population as well as in patients aged ≤ 75 years (Figures 4 and 5). The impact of current alcohol consumption on CS in patients with AF also remains controversial. A previous report suggests that current alcohol consumption may elevate the risk of ischemic stroke in patients with newly diagnosed AF, whereas abstaining from alcohol after AF diagnosis appears to mitigate this risk. These observations underscore the potential benefits of lifestyle interventions, including moderation of alcohol intake, as part of a holistic strategy for AF management to improve clinical outcomes [18]. Conversely, in the other report, the results did not demonstrate a significant relationship between low to moderate alcohol consumption and the risk of stroke or other cardiovascular events, indicating that specific recommendations regarding alcohol intake may not be warranted in AF patients [19]. Alcohol intake can affect hemostatic processes, including coagulation, fibrinolysis, and platelet function. Excessive alcohol consumption has been linked to a procoagulant state along with reduced fibrinolytic capacity [20], both of which may increase susceptibility to thrombotic events [21]. Furthermore, ethanol can amplify inflammatory responses through direct metabolic effects and activation of Kupffer cells [22]. In turn, inflammation can enhance coagulation, while coagulation processes can reciprocally influence inflammatory activity [23]. In the present study, we demonstrated that daily alcohol consumption was a significant risk factor for the development of CS in patients aged ≤ 75 years. These findings suggest that, particularly among younger patients with a relatively low burden of conventional embolic risk factors, the potential contribution of daily alcohol consumption to thromboembolic events should be carefully considered. Similar to the analysis for smoking, these findings should be interpreted with caution, as the inpatient case–control design and potential differences in prior clinical management and healthcare exposure may have influenced both alcohol consumption and the occurrence of cardioembolic stroke, limiting causal inferences. In contrast, daily alcohol consumption was not identified as a significant predictor of CS among AF patients aged ≥ 75 years. This finding may be attributable to the stronger influence of age‐related factors, such as a higher burden of comorbidities, increased frailty, and age‐associated changes in vascular and hemostatic function, which may outweigh the impact of alcohol consumption on thromboembolic risk in this older population.

In Table 1, daily alcohol consumption was more common in patients without CS. However, both factors were independently associated with events in the multivariate analysis. This discrepancy may be explained by confounding, as daily drinkers may differ from non‐drinkers in age (younger), sex (male), and comorbidities. Adjustment for these covariates likely clarified the independent associations of daily alcohol consumption with CS.

### 4.4 Clinical Implication

The present findings indicate that current smoking and daily alcohol consumption are associated with an increased risk of thromboembolic events in patients with AF, particularly among those aged ≤ 75 years. Numerous risk stratification models have been proposed to estimate embolic risk in patients with AF, including the CHADS_2_, CHA_2_DS_2_‐VASc, CHA_2_DS_2_‐VA, and HELT‐E_2_S_2_ scores [1, 6, 24, 25]. Notably, the HELT‐E_2_S_2_ score incorporates low body weight as a risk factor, which is consistent with the results of our multivariable analyses. However, several components such as sex and diabetes are not consistently weighted across these scoring systems. Our age‐stratified analyses further suggest that the relative contribution of individual risk factors may differ according to age. These findings highlight the potential value of incorporating age‐specific weighting and lifestyle‐related factors, such as smoking and alcohol consumption, into embolic risk assessment. Refining existing risk scores or developing novel, age‐adapted scoring systems that better reflect contemporary patient characteristics may improve risk stratification and support more personalized management strategies for patients with AF. Furthermore, these results highlight the potential importance of discussing the need for anticoagulant therapy based on age‐stratified embolic risk scores in patients with AF, enabling more precise identification of individuals who would benefit from anticoagulation while minimizing unnecessary exposure in those at lower risk.

### 4.5 Study Limitation

This study has several limitations. First, the cross‐sectional design employed to assess the association between smoking, alcohol consumption, and prevalent AF, with CS as the primary outcome, precludes determination of a temporal or causal relationship between current smoking or daily alcohol intake and CS. This design may also introduce survival bias, as individuals with both smoking or high alcohol consumption and AF who experienced adverse outcomes could be underrepresented. Second, the study utilized an inpatient database, which may be subject to selection bias, limiting the generalizability of findings to the broader population. The cross‐sectional nature of the dataset precluded longitudinal analyses, such as evaluating the association between smoking or alcohol consumption and mortality. In addition, both smoking and alcohol intake were reported as lifetime averages at the time of admission, making it impossible to confirm whether these exposures preceded AF onset, and thereby the temporal relationship between exposure and outcome remains uncertain. The database lacks information on outpatient medication use prior to hospitalization, including anticoagulants. Patients in the AF without CS group may have had greater prior exposure to medical care and anticoagulation, whereas those in the AF with CS group may have had lower rates. These differences could have influenced the occurrence of CS and clinical characteristics at admission, but could not be directly evaluated, representing potential residual confounding. In addition, the ICOD‐R database is based on the WHO ICD‐10 diagnostic classification and does not include procedure‐specific codes for electrophysiological interventions such as AF catheter ablation. Therefore, prior ablation history could not be reliably identified. Although this indirect analysis between institutions performing AF catheter ablation and those not performing ablation cannot exclude residual confounding at the individual patient level, the findings suggest that the observed associations were not solely explained by differences in institutional AF catheter ablation status. Nevertheless, the observed heterogeneity in effect size between institutional strata indicates that differences in patient composition and healthcare utilization may still have influenced the magnitude of the associations. In addition, individual information on prior rhythm‐control strategies, outpatient management, healthcare exposure, and lifestyle interventions was unavailable. Therefore, residual confounding related to differences in healthcare utilization and treatment history cannot be completely excluded. Although some cardiovascular comorbidities were more prevalent in the AF without CS group, their low frequency (~15% for heart failure) limits interpretation. Third, due to limitations in the ICOD‐R dataset, adjustment for factors other than age, hypertension, and diabetes such as laboratory parameters was not possible. Subgroup analyses were exploratory and included analyses using the 75‐year age threshold, which was not pre‐specified. Multiple comparisons were not formally adjusted, which may increase the risk of Type I error. Sensitivity analyses using alternative age cutoffs and additional adjustments were performed to assess the robustness of the findings. Nevertheless, these results should be interpreted cautiously, considering the potential for inflated false‐positive findings. Fourth, the ICOD‐R dataset is fully anonymized, making it impossible to perform chart reviews or other validations to confirm the diagnostic accuracy of AF. Finally, due to differences in prior clinical management, healthcare exposure, and hospitalization pathways, as well as unmeasured confounders, the observed association between smoking and CS cannot be interpreted as causal.

## 5 Conclusion

This large cohort study of patients with AF demonstrated that current smoking and daily alcohol consumption are independently associated with an increased risk of CS, particularly in individuals aged ≤ 75 years. Younger patients with a lower burden of traditional embolic risk factors appear to be especially susceptible to the thromboembolic effects of these lifestyle factors. Our findings highlight the importance of considering age‐specific contributions of modifiable lifestyle behaviors in risk assessment and management of AF. Incorporating smoking and alcohol habits into embolic risk stratification may enhance personalized preventive strategies and improve clinical outcomes in patients with AF.

## Funding

The authors have nothing to report.

## Conflicts of Interest

The authors declare no conflicts of interest.

## Supporting information


**Figure S1:** Multivariable logistic regression analysis for CS in institutions performing AF catheter ablation illustrated as Forest plot. CI, confidence interval; CS, cardioembolic stroke.

**Figure S2:** Multivariable logistic regression analysis for CS in institutions not performing AF catheter ablation illustrated as Forest plot. CI, confidence interval; CS, cardioembolic stroke.

**Table S1:** Comparison of smoking and alcohol consumption between institutions performing and not performing AF catheter ablation, stratified by the presence or absence of CS.

## Data Availability

Our study data will not be made available to other researchers because of restrictions of the Inpatient Clinico‐Occupational Database of Rosai Hospital Group.
