# Supplementary figures and images for: Lifestyle Factors and Thromboembolic Risk in Atrial Fibrillation: Age‐Dependent Effects of Smoking and Alcohol Consumption
Source: J Arrhythm. 2026 Jul 7;42(4):e70413. doi: 10.1002/joa3.70413 (PMC13338704; doi:10.1002/joa3.70413)

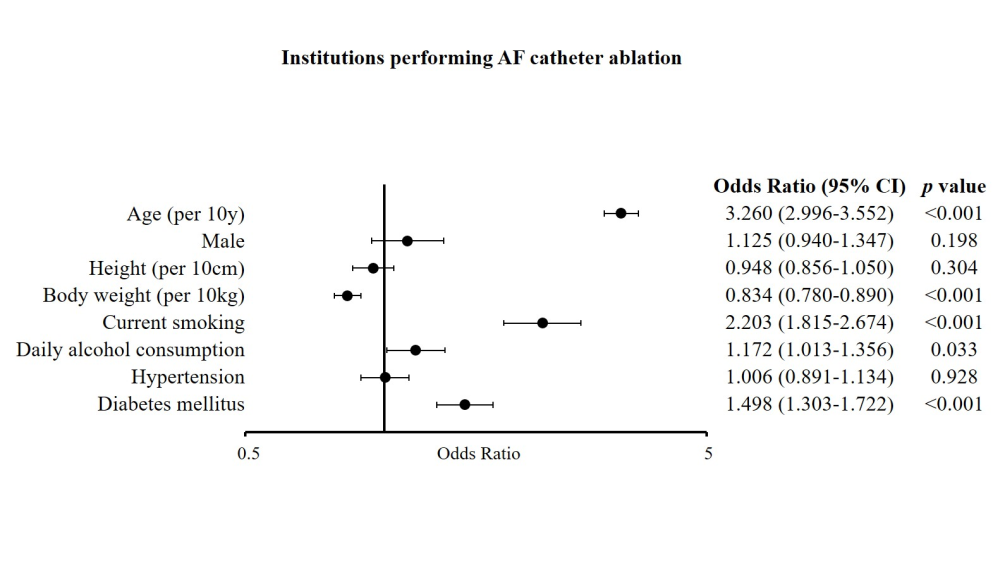

Supplement: Supplementary file 1 — Figure S1: Multivariable logistic regression analysis for CS in institutions performing AF catheter ablation illustrated as Forest plot. CI, confidence interval; CS, cardioembolic stroke. [file JOA3-42-e70413-s002.tif]

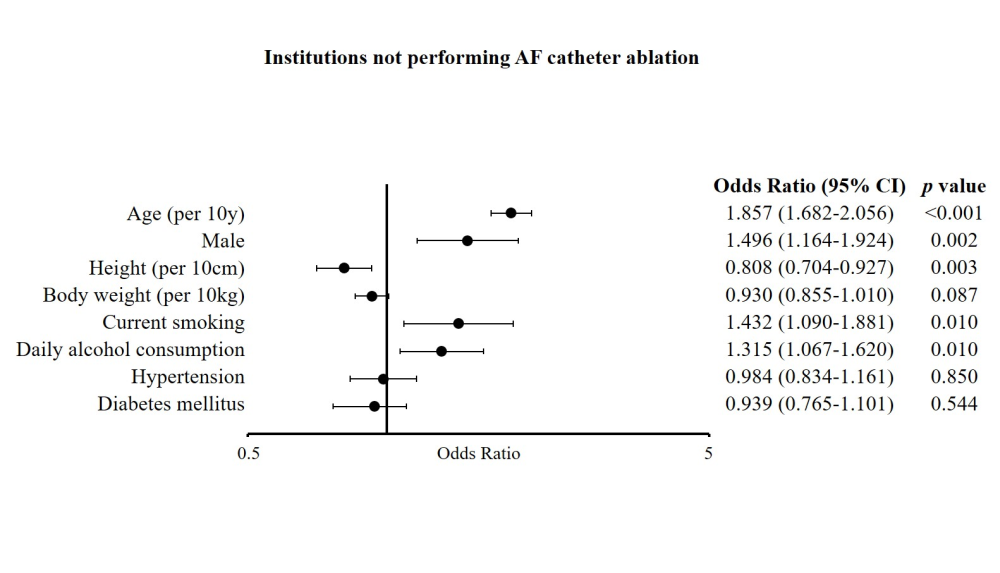

Supplement: Supplementary file 2 — Figure S2: Multivariable logistic regression analysis for CS in institutions not performing AF catheter ablation illustrated as Forest plot. CI, confidence interval; CS, cardioembolic stroke. [file JOA3-42-e70413-s003.tif]
