# Supplementary material for: Lifestyle Factors and Thromboembolic Risk in Atrial Fibrillation: Age‐Dependent Effects of Smoking and Alcohol Consumption
Source: J Arrhythm. 2026 Jul 7;42(4):e70413. doi: 10.1002/joa3.70413 (PMC13338704; doi:10.1002/joa3.70413)
Supplement: Supplementary file 3 — Table S1: Comparison of smoking and alcohol consumption between institutions performing and not performing AF catheter ablation, stratified by the presence or absence of CS. [file JOA3-42-e70413-s001.docx]

**Supplementary Table 1** Comparison of smoking and alcohol consumption between institutions performing and not performing AF catheter ablation, stratified by the presence or absence of CS

|  | **AF without CS (n=21227)** | | **P value** | **AF with CS (n=5688)** | | **P value** |
| --- | --- | --- | --- | --- | --- | --- |
|  | **Ablation (+) hospitals**  **(10 hospitals, n=16346)** | **Ablation (-) hospitals**  **(22 hospitals, n=4881)** |  | **Ablation (+) hospitals**  **(10 hospitals, n=2817)** | **Ablation (-) hospitals**  **(22 hospitals, n=2871)** |  |
| **Smoking** |  |  |  |  |  |  |
| **Former/Current** | 8925 (54.6) | 2355 (48.3) | <0.001 | 1150 (40.8) | 1097 (38.2) | 0.044 |
| **Current (daily)** | 2099 (12.8) | 657 (13.5) | 0.259 | 348 (12.4) | 392 (13.7) | 0.145 |
| **Drinking** |  |  |  |  |  |  |
| **Former/Current** | 10264 (62.8) | 2663 (54.6) | <0.001 | 1371 (48.7) | 1396 (48.6) | 0.973 |
| **Current (daily)** | 5468 (33.5) | 1213 (24.9) | <0.001 | 681 (24.2) | 681 (23.7) | 0.688 |

Continuous data are presented as the median (interquartile range). Categorical variables are presented as numbers (percentage). AF, atrial fibrillation; CS, cardioembolic stroke.
